# Fractional Physics Informed Neural Networks for Surrogate Modeling of Non-Markovian Discrete-Time Quantum Walks

**DOI:** 10.3390/e28080844

**Published:** 2026-07-29

**Authors:** Zhaoyu Zhu, Mingxin Liu, Chengtian Liang, Fan Yang, Jizhong Shen, Xintong Wen, Lijiong Shen, Yu Wang

**Affiliations:** 1 School of Physics, Hangzhou Normal University, Hangzhou 311121, China; 2 School of Mathematics, Hangzhou Normal University, Hangzhou 311121, China

**Keywords:** physics-informed neural networks, quantum random walks, fractional diffusion, non-Markovian processes, surrogate modeling, anomalous diffusion, 03.67.-a, 05.40.Fb, 02.60.Lj, 07.05.Mh

## Abstract

Predicting anomalous diffusion in quantum walks with non-Markovian environmental noise is computationally demanding. We introduce FracPINN, a fractional physics-informed neural network that embeds a fully differentiable, PyTorch-based Caputo PDE solver into a classical LSTM encoder. Rather than replacing classical predictors, FracPINN acts as a compact physics regularizer that constrains the inference with emergent fractional transport dynamics; every physics-informed loss component is strictly label-free, and the ground-truth exponent enters only through an explicitly supervised regression term. Evaluated on 3709 non-Markovian DTQW simulations with exponentially correlated Gaussian coin noise (filtered from 5000 raw samples to the physically admissible exponent range), FracPINN achieves a mean absolute error of 0.214 and $R^{2}=0.682$ on held-out test data, outperforming classical baselines by 5.3% in terms of the MAE overall, while adding only four interpretable physical parameters. Notably, gains concentrate in the sub-diffusive regime where memory effects dominate, with a 13.5% MAE improvement over the baseline there, yet the normal and super diffusive accuracy remains intact. Once trained, the surrogate reduces inference from seconds of simulation to fractions of a millisecond per sample. These results show that our fractional PDE networks are most compelling as targeted physics refinements with minimal overhead within stable classical pipelines.

## 1. Introduction

Quantum random walks (QRWs) provide a fundamental framework for understanding quantum transport and have been identified as a key primitive for quantum algorithms, quantum simulation, and quantum-enhanced search protocols [1,2,3]. Unlike their classical counterparts, discrete-time quantum walks (DTQWs) exhibit ballistic spreading in idealized settings, a consequence of quantum interference between amplitudes on a lattice [4]. However, real-world implementations are inevitably coupled to environmental degrees of freedom, leading to decoherence and modified transport regimes that range from ballistic to diffusive and even to localization [5,6,7].

A particularly important class of environmental noise arises when the system bath coupling exhibits temporal correlations, or the so-called non-Markovian noise. In such settings, the quantum walker’s dynamics retain a memory of past states, and the resulting spatial probability distribution may exhibit anomalous diffusion, characterized by a mean-squared displacement (MSD) that scales as $\langle x^{2}\left(t\right)\rangle ∼t^{\alpha }$ with α≠1 [8,9]. The parameter α serves as an effective order parameter for the transport regime; α<1 corresponds to sub-diffusion (memory trapping), α≈1 corresponds to normal diffusion, and α>1 corresponds to super diffusion or persistent acceleration [10,11,12]. Experimental platforms including trapped ion systems [5] and photonic waveguides [13] have demonstrated non-Markovian effects, yet the theoretical characterization of these regimes remains computationally expensive, typically requiring repeated simulation of the full unitary evolution followed by statistical averaging.

Recent advances in physics-informed machine learning [14,15] offer a promising alternative. Physics-informed neural networks (PINNs) embed governing differential equations directly into the loss function of a neural network, thereby constraining the learned solution to be physically consistent [16,17]. While PINNs have been successfully applied to integer-order PDEs in fluid mechanics [16], heat transfer [18], and orbital mechanics [19], their extension to fractional order systems is less developed. Fractional PINNs (fPINNs), introduced by Pang et al. [20], incorporate Caputo or Riesz fractional derivatives into the neural network loss, enabling the solution of forward and inverse problems for anomalous diffusion [21,22]. However, existing fPINN frameworks have focused on direct solution of the fractional PDE from sparse data or known boundary conditions; they have not been applied to the more challenging inverse problem of inferring fractional parameters from coarse-grained observables of an underlying quantum stochastic process.

In parallel, machine learning methods for anomalous diffusion classification have emerged, with recurrent neural networks (RNNs) and convolutional networks being trained to segment single-particle trajectories into normal, sub-diffusive, and super diffusive categories [23,24,25,26,27]. These approaches are primarily data-driven and do not explicitly enforce the fractional transport physics governing the dynamics. Moreover, they focus on the trajectory level rather than the mesoscopic statistical regime relevant to quantum walk ensembles.

The present approach occupies a complementary niche to the works cited above, with the following concrete advantages. (1) Compared with direct DTQW simulation, the surrogate amortizes the cost of the full unitary evolution. Once trained, parameter inference for a new sample requires only a fraction of a millisecond instead of seconds to minutes of simulation per noise realization, which makes large parameter sweeps and interactive calibration practical (Section 5.5). (2) Compared with purely data-driven trajectory classifiers [23,24,25,26,27], our model operates on cheap, coarse-grained ensemble statistics rather than raw trajectory data, and it embeds the fractional transport equation as a structural prior. As shown in Section 5.3, this prior yields its largest accuracy gain precisely in the memory-dominated sub-diffusive regime, where purely data-driven regression is least reliable. (3) Compared with existing fPINN formulations [20,21,22], which solve the fractional PDE forward in time from known coefficients or boundary data, we address the inverse problem of inferring the effective fractional parameters of an underlying quantum stochastic process from late-time observables. To the best of our knowledge, this is the first fractional PINN surrogate for non-Markovian quantum walks.

In this work, we connect fractional physics-informed learning to quantum walk simulation by introducing FracPINN, a surrogate model that infers the effective anomalous diffusion exponent and fractional transport parameters from coarse-grained statistical features of non-Markovian DTQW simulations. Our contributions are threefold. Firstly, we formulate the inverse problem of inferring fractional diffusion parameters from reduced observables (variance, entropy, kurtosis, and spatial correlation) extracted from DTQW simulations with exponentially correlated Gaussian coin noise. Secondly, we design a neural architecture combining an LSTM encoder for time series statistics with a fully differentiable fractional PDE solver based on the Caputo derivative. The model is trained with a four-component loss function comprising data consistency, differentiable solver consistency, physical regularization, and supervised exponent regression. Crucially, none of the physics-informed loss components use the ground-truth exponent; the label enters only through the explicitly supervised regression term. What is more, we conduct extensive experiments on a dataset of 3709 physically admissible DTQW simulations spanning sub-diffusive, normal, and super diffusive regimes. Our model achieves an MAE of 0.214 and $R^{2}=0.682$, outperforming the baseline MLP by 5.3% in terms of MAE. Ablation studies confirm that the physics-informed loss terms are most beneficial in the sub-diffusive regime.

The rest of this paper is organized as follows. Section 2 formulates the mathematical problem. Section 3 describes the DTQW simulator, coarse-grained statistics, fractional PDE solver, and FracPINN architecture. Section 4 details the experimental set-up and dataset generation. Section 5 presents the main results, ablation study, and regime analysis. Section 6 discusses implications and limitations, and Section 7 concludes the paper.

## 2. Problem Formulation

We consider a one-dimensional discrete-time quantum walk on a lattice x∈Z with a coin degree of freedom. At each time step *t*, the joint state of the walker and coin is evolved by a position shift operator and a coin rotation:

(1) $$|\Psi \left(t+1\right)\rangle =S\cdot C\left(\theta_{t}\right)\;|\Psi \left(t\right)\rangle ,$$

where *S* shifts the walker conditioned on the coin state and $C(\theta_{t})$ applies a rotation of an angle $\theta_{t}$ to the coin. In the presence of environmental noise, the coin phase becomes a stochastic process. For non-Markovian colored Gaussian noise, we model the phase sequence $\left\{\phi_{t}\right\}$ as a stationary Gaussian process with zero mean and exponential covariance:

(2) $$\langle \phi_{t}\phi_{t^{\prime }}\rangle =\sigma^{2}\exp\;\left(-|t-t^{\prime }|/\tau_{c}\right),$$

where σ is the noise strength and $\tau_{c}$ is the correlation time. Equivalently, in the discrete time $\phi_{t}$ is an AR(1) or Ornstein–Uhlenbeck process with a correlation coefficient $\rho =e^{-1/\tau_{c}}$. We sample the joint sequence directly from the Gaussian process via the Cholesky factorization of the covariance matrix [28]. This exponentially correlated process introduces temporal memory into the coin dynamics, which propagates into the walker’s spatial distribution.

After *T* steps, the spatial probability distribution ρ(x,t) is extracted from each realization. The resulting distribution exhibits anomalous diffusion characterized by an effective exponent α such that

(3) $$\langle x^{2}\left(t\right)\rangle ∼t^{\alpha }.$$

Our goal is to learn a surrogate model $f_{\theta }$ that maps a vector of coarse-grained statistical features s(t), extracted from the early time ($t\leq T_{\mathrm{early}}$) evolution, to the effective diffusion exponent:

(4) $$\hat{\alpha }=f_{\theta }\left(s(t)\right).$$

Rather than treating this as a purely data-driven regression, we embed physical constraints through a fractional diffusion model. Specifically, we assume that the mesoscopic density ρ(x,t) satisfies a generalized fractional diffusion equation with a memory kernel:

(5) $$\frac{\partial^{\beta }\rho }{\partial t^{\beta }}=D_{0}\;\Delta \rho +\int_{0}^{t}K\left(t-\tau \right)\;\Delta \rho \left(x,\tau \right)\;d\tau ,$$

where $\partial^{\beta }/\partial t^{\beta }$ denotes the Caputo fractional derivative of the order β with β=α/2, $D_{0}$ is a generalized diffusion coefficient, and the memory kernel $K\left(\tau \right)=\gamma \;e^{-\tau /\tau_{m}}$ captures the non-Markovian character of the environment, while γ is the kernel strength and $\tau_{m}$ the memory decay time. The spatial operator Δ is the standard Laplacian on the one-dimensional lattice.

On the choice of the Caputo derivative, several definitions of the fractional derivative exist, and we use the Caputo derivative [29] for the following reasons. (1) Regarding the initial conditions, the Caputo derivative admits standard integer-order initial conditions (ρ(x,0), and $\partial_{t}\rho \left(x,0\right)$ when β>1), whereas the Riemann–Liouville derivative requires fractional-order initial data that lack a direct physical interpretation for an evolving probability density. (2) For causality, the Caputo operator accumulates memory from t=0 onward, matching the causal build-up of correlations in the driven quantum walk. (3) Finally, for numerical maturity, the L1 discretization of the Caputo derivative is well studied and conditionally stable for 0<β≤1 [30], which is precisely the order range used here. The main drawbacks are the weakly singular kernel at t=0, which reduces the accuracy at early times, the O(n) history cost per step, and the limited accuracy order $O(\Delta t^{2-\beta })$ of the L1 scheme. We quantify the associated discretization error by grid refinement in Section 3. The Grünwald–Letnikov definition is equivalent to the Caputo one for sufficiently smooth functions vanishing at t=0 but leads to less stable weight recurrences, while the Riesz derivative acts on space rather than time and is therefore not suited to the temporal memory studied here.

On the relation β=α/2, the fractional order β predicted by the network is related to the MSD exponent by α=2β for the following reasons. (1) Coverage and numerical stability, as for the underlying unit-step lattice walk, the MSD is bounded by the ballistic limit α∈(0,2], and the map β=α/2 sends this entire physical range onto (0,1], exactly the interval in which the Caputo L1 scheme is defined and conditionally stable [30]. (2) Regarding Hurst index interpretation, the exponentially correlated coin noise acts as a persistent random drive in analogy with fractional Brownian motion, whose MSD scales as $t^{2H}$ with the Hurst index H∈(0,1) [31]; identifying β with *H* yields α=2β, with β<1/2, β≈1/2, and β>1/2 corresponding to sub-, normal, and super diffusion, respectively. This identification is used consistently in the power-law proxy $\langle x^{2}\rangle =D_{0}t^{2\beta }$ of the data loss (Section 3). (3) Finally, there is the role of the memory kernel. The kernel $K\left(\tau \right)=\gamma e^{-\tau /\tau_{m}}$ in Equation (5) is not intended to determine the asymptotic order; it models short- and intermediate-time deviations from ideal power-law scaling (crossover behavior), while the asymptotic exponent is carried by the Hurst-like parameter β. We emphasize that Equation (5) is an effective mesoscopic description; in the pure Caputo diffusion limit (γ→0), its MSD scales as $t^{\beta }$ asymptotically, and thus Equation (5) should be read as a physics-based regularizer of the variance curve shape rather than as a first-principles derivation of the exponent mapping. The exponent mapping itself is calibrated through the supervised term (Section 3).

The inverse problem is to infer the parameters $\left\{\beta ,\gamma ,\tau_{m},D_{0}\right\}$ from the observable statistics s(t), from which α=2β follows directly. We emphasize that this is an effective description; the fractional PDE is a mesoscopic approximation of the underlying microscopic quantum dynamics, and the parameters are emergent rather than fundamental. They summarize the transport behavior of the underlying quantum dynamics at the mesoscopic level.

## 3. Methods

Our simulator implements a coined DTQW on a one-dimensional lattice of 401 sites (x∈[−200,200]) with a two-dimensional coin space {|L〉,|R〉}. The shift operator is defined as follows:

(6) $$S=\sum_{x}\left(|x+1\rangle \langle x|⊗|R\rangle \langle R|+|x-1\rangle \langle x|⊗|L\rangle \langle L|\right).$$

The coin rotation at each step is

(7) $$C\left(\theta_{t}\right)=e^{-i\theta_{t}\sigma_{y}},$$

with $\sigma_{y}$ being the Pauli-*y* matrix. The colored noise is generated as follows. For each sample, we draw the noise strength σ from a Beta(2,2) distribution on [0,1.5] and the correlation time $\tau_{c}$ uniformly from [0,20] (Section 4.1), and we sample the phase sequence ${\left\{\phi_{t}\right\}}_{t=1}^{T}$ from the Gaussian process in Equation (2) via Cholesky factorization of the T×T covariance matrix. The noise enters the coin as a random phase $C_{t}=\mathrm{diag}\left(e^{i\phi_{t}/2},e^{-i\phi_{t}/2}\right)\;C\left(\theta \right)$ with a fixed base coin angle θ per sample. The initial walker state is localized at x=0 with coin state |R〉.

**Figure 1:**
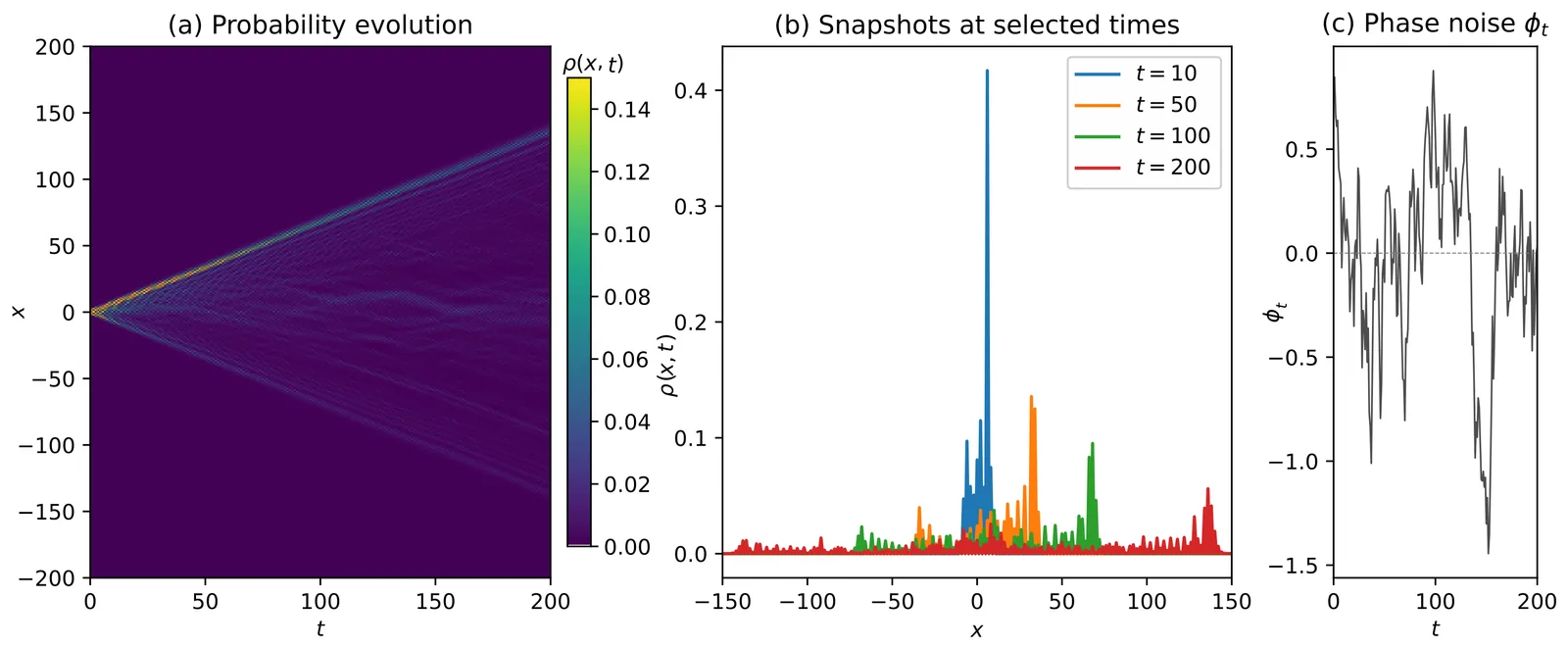
(**a**) Spatiotemporal probability evolution of a non-Markovian DTQW. (**b**) Snapshots of the spatial distribution at selected times. (**c**) The exponentially correlated Gaussian phase noise $\phi_{t}$ generated from the covariance function in Equation (2).

For each sampled parameter triple $\left(\theta ,\sigma ,\tau_{c}\right)$, we simulate one noise realization up to T=500 steps, and the coarse-grained statistics (Section 3) are computed from the spatial probability distribution of that realization. Two comments on this protocol are in order. First, T=500 is required by the downstream task itself; the target exponent α is fitted via log-log regression of the MSD over the asymptotic window t∈[100,500], and thus the trajectory must extend well beyond the transient. The early-time input to the network uses only $t\leq T_{\mathrm{early}}=10$. Second, we use a single realization per parameter set rather than an ensemble average over *M* realizations for two reasons: (1) the spatial distribution of a single realization already provides a smooth statistical object from which the four coarse-grained observables are well defined, and the exponent fit aggregates 400 time points; and (2) the computational cost scales linearly with *M*. A single 500-step realization costs ≈0.23 s on our workstation (Section 4.2), and thus generating the 5000 raw samples with M=1 takes ≈20 CPU minutes, whereas M=50 would take ≈16 CPU-hours. Averaging over M=50 realizations would reduce the variance in the per-sample statistics, but it would not change the quantity being estimated (the underlying anomalous exponent), and the single-realization protocol proved sufficient for the accuracy levels reported in Section 5. Figure 1 shows a representative probability evolution and the associated coin noise trajectory.

From the spatial probability matrix ρ(x,t) of each realization, we extract a four-dimensional statistical feature vector at each time step:

1. **Variance:** $\langle x^{2}\left(t\right)\rangle =\sum_{x}x^{2}\rho \left(x,t\right)$.
2. **Shannon entropy:** $S\left(t\right)=-\sum_{x}\rho \left(x,t\right)\ln\rho \left(x,t\right)$, normalized by $\ln N_{x}$.
3. **Kurtosis:** $\kappa \left(t\right)=\langle x^{4}\left(t\right)\rangle /{\langle x^{2}\left(t\right)\rangle }^{2}$, evaluated on the centered distribution.
4. **Spatial correlation:** $C\left(t\right)=\sum_{x}{\left[\rho \left(x+1,t\right)-\rho \left(x,t\right)\right]}^{2}$, a nearest-neighbor difference measure that is large for localized (rough) distributions.

**Figure 2:**
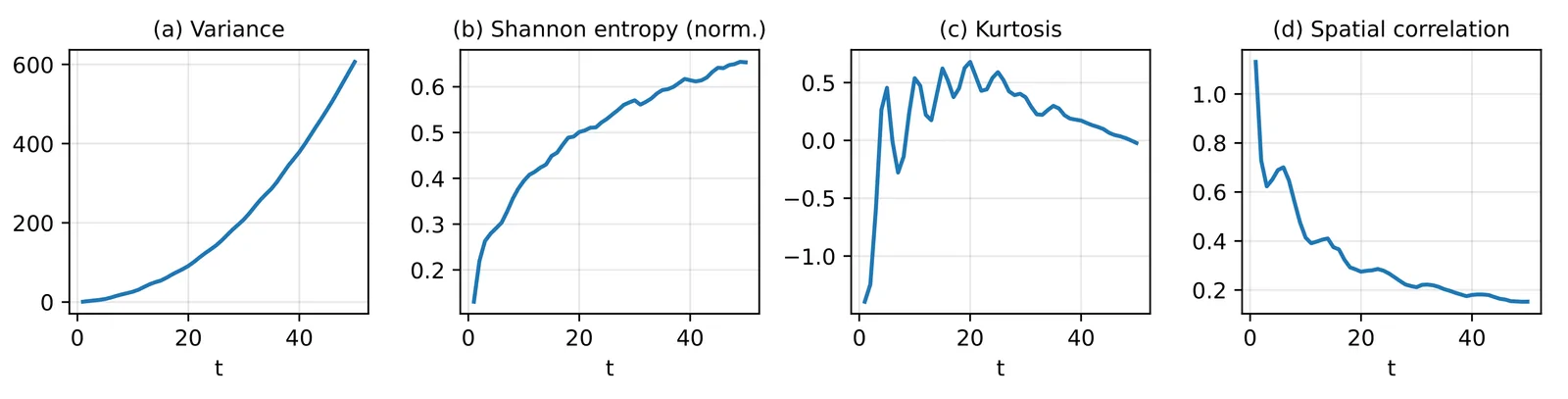
Coarse-grained statistical observables extracted from a representative DTQW sample: (**a**) variance, (**b**) Shannon entropy, (**c**) kurtosis, and (**d**) spatial correlation. All quantities are shown as functions of the time *t*.

These statistics are normalized to a zero mean and unit variance using a StandardScaler fitted on the training set. The early time window $t\in [1,T_{\mathrm{early}}]$ with $T_{\mathrm{early}}=10$ provides the input to the neural network. Figure 2 illustrates the four statistics for a representative sample.

The fractional PDE (Equation (5)) is solved numerically on a one-dimensional lattice with spacing Δx=1 and a time step Δt=1. The Caputo time fractional derivative of the order β is discretized using the L1 scheme [30]:

(8) $$\frac{\partial^{\beta }\rho }{\partial t^{\beta }}{|}_{t_{n}}\approx \frac{\Delta t^{-\beta }}{\Gamma (2-\beta )}\sum_{k=0}^{n-1}c_{k}\left(\beta \right)\;\left(\rho_{n-k}-\rho_{n-k-1}\right),$$

with coefficients $c_{k}\left(\beta \right)={\left(k+1\right)}^{1-\beta }-k^{1-\beta }$, and $c_{0}=1$. Solving Equation (8) for $\rho_{n}$ yields the explicit update

(9) $$\rho_{n}=\rho_{n-1}+\Gamma \left(2-\beta \right)\;\Delta t^{\beta }\left(D_{0}\Delta \rho_{n-1}+M_{n}\right)-\sum_{k=1}^{n-1}c_{k}\left(\beta \right)\left(\rho_{n-k}-\rho_{n-k-1}\right),$$

which reduces to the classical explicit Euler scheme for β=1.

The spatial Laplacian is discretized by the standard three-point stencil with zero-flux boundary conditions, and the memory integral is computed via trapezoidal quadrature with the kernel $K\left(\tau \right)=\gamma e^{-\tau /\tau_{m}}$.

Three considerations motivate the unit discretization. (1) For physical matching, the surrogate PDE is defined on the same lattice as the DTQW itself; the walker moves by exactly one site per step, and thus Δx=1 and Δt=1 are the native resolutions of the microscopic dynamics. A finer grid would resolve sub-lattice scales on which no physical information exists. (2) Regarding stability, the explicit update in Equation (9) requires $D_{0}\;\Delta t^{\beta }/\Delta x^{2}≲1/2$; with Δx=Δt=1, this holds for $D_{0}≲0.5$, which we enforce inside the solver through the smooth saturation $D_{0}^{\mathrm{eff}}=D_{0}^{\max}\tanh\left(D_{0}/D_{0}^{\max}\right)$ with $D_{0}^{\max}=0.45$ (identity to first order near $D_{0}=0$, differentiable everywhere). (3) Finally, for the verified accuracy, we repeated the solution on refined grids (Δx,Δt)=(0.5,0.15) and (0.25,0.02), both inside the stability region, for the representative parameters (β∈{0.5,0.8,1.0}, γ∈{0,0.3,0.2}, $\tau_{m}=5$, and $D_{0}=0.3$). Relative to the finest grid, the unit grid reproduces the variance curve within the early training window t≤10 with a normalized-shape deviation of ≤2% for β≥0.8; only for deep sub-diffusion (β=0.5), where the $t^{-\beta }$ kernel singularity is strongest, does the local shape deviation reach ≈20%. Since the training losses compare normalized variance shapes (Section 3) and the supervised term dominates the exponent regression, this level of discretization error is acceptable for the present surrogate; implicit or adaptively refined solvers are left to future work (Section 6).

Two implementations of the same scheme were used. For training, the solver was written entirely in PyTorch as batched tensor operations over the early window (81 spatial points, t≤10); the L1 coefficients (Equation (8)) are functions of the predicted β, the memory convolution involves the predicted $\left(\gamma ,\tau_{m}\right)$, and the Laplacian term involves the predicted $D_{0}$. Therefore, the full solution $\hat{\rho }\left(x,t\right)$—and any loss computed from it—is differentiable with respect to all four predicted parameters, with gradients flowing through Γ(2−β) via torch.lgamma. For post hoc analysis and figure generation outside the training loop, we used an equivalent NumPy reference implementation. This resolves the solver into the training pipeline without third-party autodiff bridges, and it renders the physics consistency loss below a genuinely differentiable quantity rather than a placeholder.

**Figure 3:**
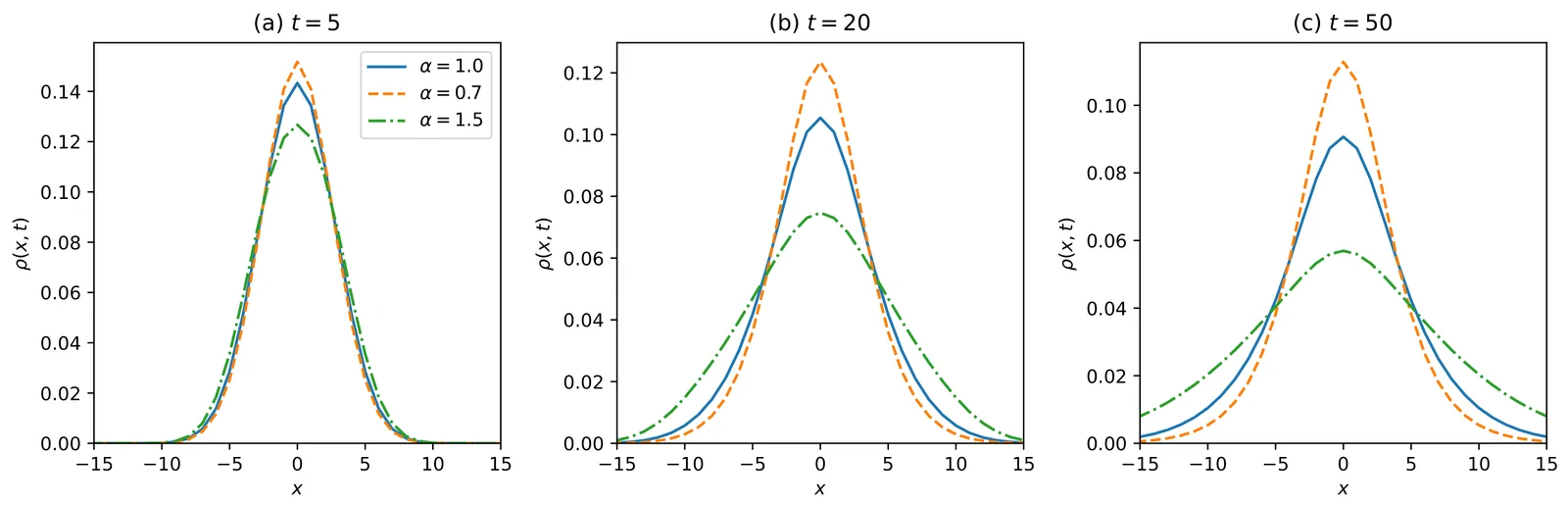
Solutions of the fractional diffusion equation for different anomalous exponents at times (**a**) t=5, (**b**) t=20, and (**c**) t=50. Solid: α=1.0 (normal); dashed: α=0.7 (sub-diffusion); dash-dotted: α=1.5 (super diffusion). The curves are true numerical solutions of Equation (5) matched with the differentiable L1 solver on the unit grid (Δx=Δt=1) at the model mapping β=α/2, with $D_{0}=0.3$, γ=0.3, and $\tau_{m}=5$.

**Figure 4:**
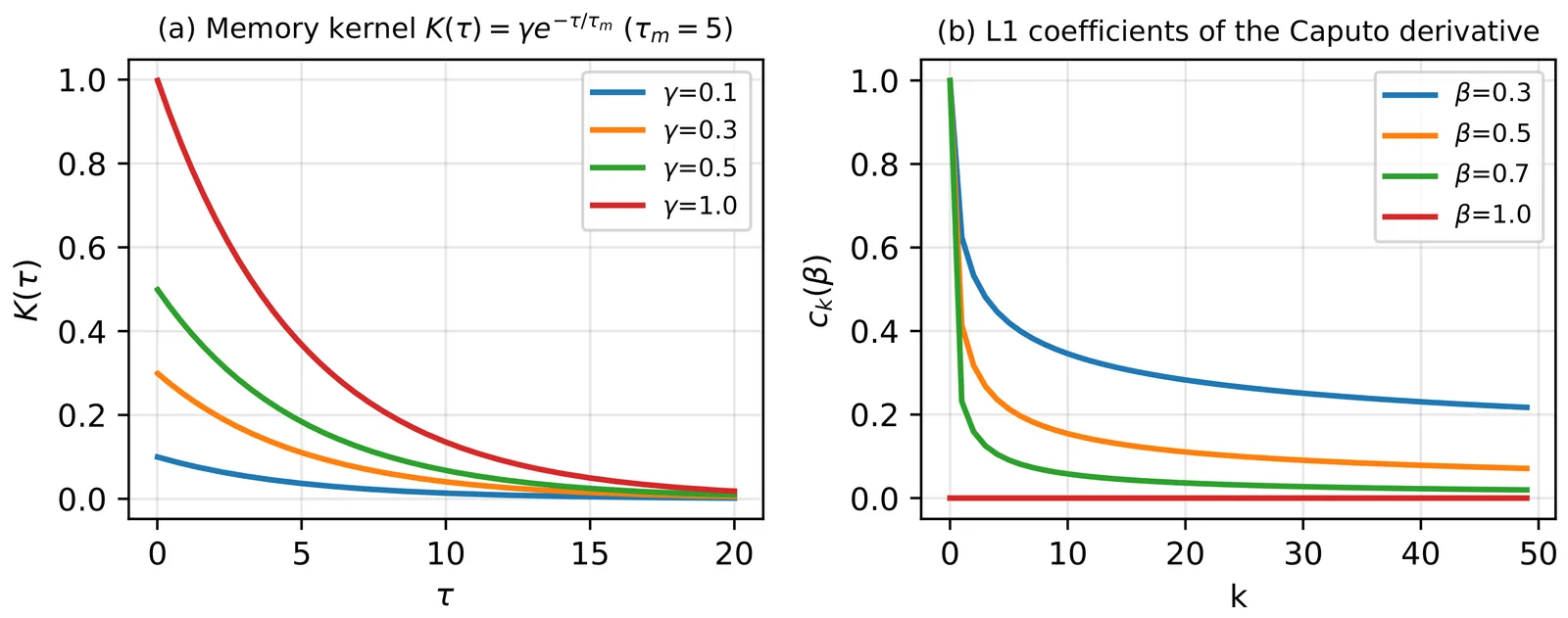
(**a**) Exponential memory kernel $K\left(\tau \right)=\gamma e^{-\tau /\tau_{m}}$ for different kernel strengths γ. (**b**) L1 coefficients $c_{k}\left(\beta \right)$ of the Caputo discretization (Equation (8)) for different fractional orders β.

During training, the FracPINN predicts the parameters $\left\{\beta ,\gamma ,\tau_{m},D_{0}\right\}$, which are fed into the solver to produce a predicted density evolution $\hat{\rho }\left(x,t\right)$. This density is then used to compute the physics consistency loss. Representative solutions of the fractional diffusion equation for different exponents are shown in Figure 3, and the memory kernel together with the Caputo derivative weights is visualized in Figure 4.

The FracPINN model consists of two modules: a time series encoder and a proxy parameter head.

In the encoder, the input is a tensor of a shape $\left(B,T_{\mathrm{early}},4\right)$, where *B* is the batch size and 4 is the number of coarse-grained statistics. A three layer (unidirectional) LSTM with a hidden dimension of 128 processes the time sequence and produces a final hidden state $z\in R^{128}$.

For the proxy parameter head, a fully connected network with hidden layers of sizes 256, 128, and 64 maps z to the four physical parameters:

(10) $$\begin{array}{cc} \hat{\beta } & =\sigma (z_{1})\times 0.99+0.01,\\ \hat{\gamma } & =\mathrm{softplus}\left(z_{2}\right)+10^{-4},\\ {\hat{\tau }}_{m} & =\mathrm{softplus}\left(z_{3}\right)+10^{-4},\\ {\hat{D}}_{0} & =\mathrm{softplus}\left(z_{4}\right)+10^{-4}. \end{array}$$

**Figure 5:**
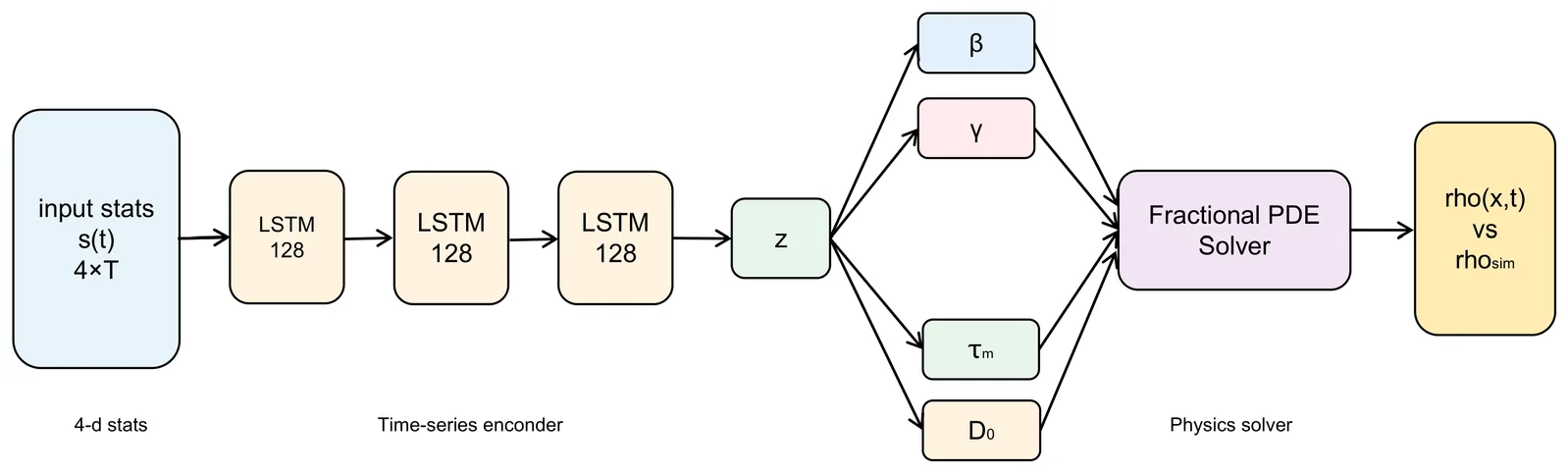
Schematic of the FracPINN architecture. The input is a time series of four coarse-grained statistics encoded via three-layer LSTM. The resulting hidden state z is mapped to four physical parameters by the proxy parameter head, which are then fed into a differentiable fractional PDE solver. The four-component loss function compares the predicted density against the simulation at the data level, solver consistency level, physical parameter level, and supervised exponent level.

The sigmoid and softplus activations enforce positivity and boundedness constraints appropriate for the physical parameters. The model has 407,236 trainable parameters. The architecture is summarized in Figure 5.

The total loss is a weighted sum of four components:

(11) $$L_{\mathrm{total}}=\lambda_{\mathrm{data}}L_{\mathrm{data}}+\lambda_{\mathrm{PDE}}L_{\mathrm{PDE}}+\lambda_{\mathrm{phys}}L_{\mathrm{phys}}+\lambda_{\sup}L_{\sup}.$$

A design principle of this work is that every physics-informed component is strictly label-free; the ground-truth exponent $\alpha_{\mathrm{true}}$ enters only through the explicitly supervised term $L_{\sup}$. This separation ensures that any benefit attributed to the fractional PDE is genuinely physics-driven rather than a disguised form of label regression.

**Data loss.** The data loss compares the predicted variance evolution from the inferred parameters with the true early time variance using the closed-form power-law proxy $v_{\mathrm{pred}}\left(t\right)=D_{0}t^{2\beta }$. Since the absolute scale depends on $D_{0}$ and is destroyed by standardization, we compare the shape of the variance curve by normalizing both the prediction and truth to the [0,1] interval:

(12) $$L_{\mathrm{data}}=\frac{1}{B}\sum_{b=1}^{B}{∥{\tilde{v}}_{\mathrm{pred}}^{\left(b\right)}-{\tilde{v}}_{\mathrm{true}}^{\left(b\right)}∥}_{2}^{2},$$

where $\tilde{v}$ denotes min-max normalization along the time axis. This formulation ensures the loss is sensitive to the exponent β rather than the absolute prefactor $D_{0}$.

**PDE-consistency loss.** The differentiable solver is marched with the predicted parameters over the early window, and the variance in the resulting density is compared with the observed early-time variance, again in a normalized shape:

(13) $$L_{\mathrm{PDE}}=\frac{1}{B}\sum_{b=1}^{B}{∥{\tilde{v}}_{\mathrm{PDE}}^{\left(b\right)}\left(\hat{\beta },\hat{\gamma },{\hat{\tau }}_{m},{\hat{D}}_{0}\right)-{\tilde{v}}_{\mathrm{true}}^{\left(b\right)}∥}_{2}^{2},$$

where $v_{\mathrm{PDE}}\left(t\right)=\sum_{x}x^{2}\hat{\rho }\left(x,t\right)$ is computed from the solver output in Equation (9). Unlike $L_{\mathrm{data}}$, which uses the asymptotic power-law proxy, this term involves the full fractional dynamics—the Caputo history weights and the memory kernel—and gradients flow through the L1 scheme to all four parameters.

**Physical regularization**. To keep the predicted parameters in physically meaningful and identifiable ranges, we apply label-free hinge penalties:

(14) $$L_{\mathrm{phys}}=\langle \mathrm{relu}{\left(\beta -0.98\right)}^{2}+\mathrm{relu}{\left(0.02-\beta \right)}^{2}\rangle +0.1\langle \mathrm{relu}{\left(\tau_{m}/T_{\mathrm{early}}-1\right)}^{2}\rangle +0.01\langle \mathrm{relu}{\left(-\gamma \right)}^{2}+\mathrm{relu}{\left(-D_{0}\right)}^{2}\rangle ,$$

where 〈·〉 denotes the batch mean. The first term keeps β away from the hard sigmoid bounds where gradients saturate; the second enforces memory-horizon identifiability, a memory time $\tau_{m}$ much longer than the observation window cannot be inferred from the input statistics; and the third is a numerical backstop for the positivity already enforced by softplus. No ground-truth quantity enters this term.

**Supervised regression loss.** The only term that uses the ground-truth label is the supervised regression of the diffusion exponent through the model relation α=2β:

(15) $$L_{\sup}=\frac{1}{B}\sum_{b=1}^{B}{\left|2{\hat{\beta }}^{\left(b\right)}-\alpha_{\mathrm{true}}^{\left(b\right)}\right|}^{2}.$$

This is the standard supervised signal of the surrogate, and we report it as such rather than as physics.

**Figure 6:**
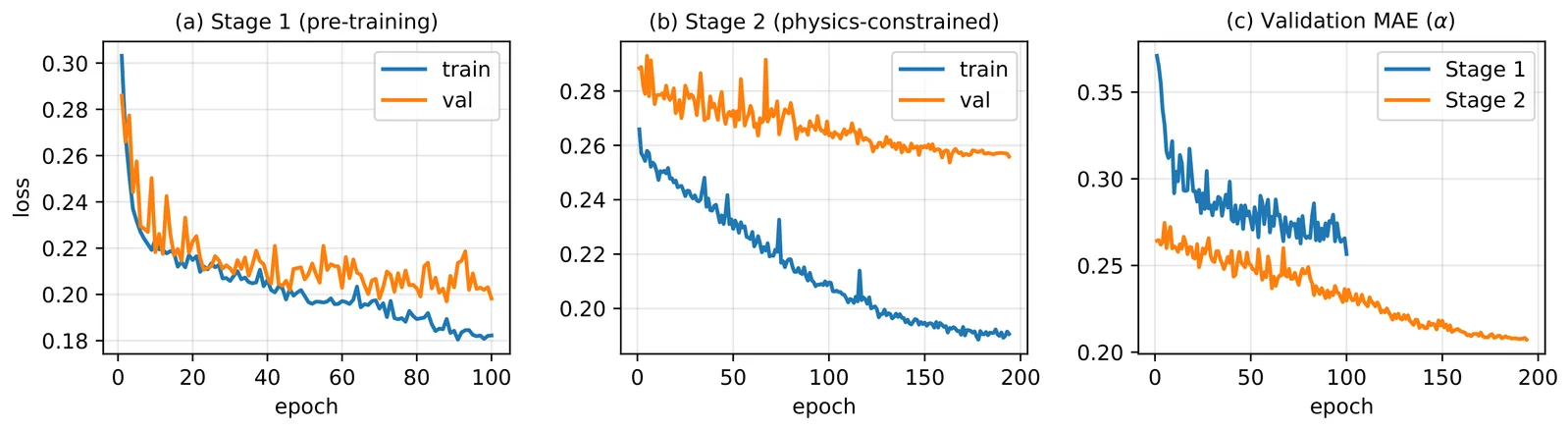
Training loss curves for (**a**) Stage 1 (pretraining) and (**b**) Stage 2 (physics-constrained training), together with the validation MAE of the diffusion exponent. (**c**) Validation MAE (α).

Training proceeds in two stages. In Stage 1 (pretraining, epochs 1–100), the differentiable-solver term is switched off ($\lambda_{\mathrm{PDE}}=0$), allowing the network to first learn the data-driven mapping from statistics to parameters through the cheap power-law proxy and the supervised term. In Stage 2 (physics-constrained, epochs 101–300), the solver term is switched on at $\lambda_{\mathrm{PDE}}=0.3$, enforcing the full fractional dynamics. The remaining weights are kept constant at $\lambda_{\mathrm{data}}=0.3$, $\lambda_{\mathrm{phys}}=0.1$, and $\lambda_{\sup}=1.0$ throughout. Both stages use the AdamW optimizer with an initial learning rate of $2\times 10^{-3}$, weight decay of $5\times 10^{-3}$, and cosine annealing to $2\times 10^{-5}$. Early stopping monitors the validation MAE with a patience of 30. The evolution of the individual loss components is shown in Figure 6.

## 4. Experimental Set-Up

### 4.1. Dataset Generation

The dataset generation pipeline consists of the following steps, which we state explicitly for reproducibility:

1. **Sampling of simulation parameters.** The simulation parameters $\left(\theta ,\sigma ,\tau_{c}\right)$—the coin angle, noise strength, and noise correlation time—were sampled over θ∈[0,π/2], σ∈[0,1.5], and $\tau_{c}\in \left[0,20\right]$. The coin angle and correlation time were drawn via Latin hypercube sampling (LHS) [32], and the noise strength from a Beta(2,2) distribution, which concentrated samples in the physically relevant moderate-noise region. Importantly, the diffusion exponent α was not a simulation input and was never sampled.
2. **Simulation.** For each parameter triple, one DTQW realization of T=500 steps was simulated as described in Section 3.
3. **A posteriori exponent fitting.** For each realization, the exponent α was fitted via log-log linear regression of the MSD over the asymptotic window t∈[100,500]. The fitted α was a derived label and not a simulation parameter.
4. **Physical-range filtering.** Samples whose fitted exponent fell outside the physically admissible range (0.05,2.0] were discarded. Negative or near-zero exponents were fitting artifacts of strongly trapped realizations whose variance was essentially flat, and thus the log-log regression reduced to fitting numerical noise; a negative MSD power-law exponent was not physical for an initially localized walker. Exponents above 2 exceeded the ballistic bound $\langle x^{2}\rangle \leq t^{2}$ of any unit-step lattice walk and corresponded to transient acceleration rather than asymptotic scaling. Of the 5000 raw samples, 642 (12.8%) were removed for α≤0.05, and 649 (13.0%) were removed for α>2, leaving 3709 physically admissible samples with α∈[0.051,2.000].
5. **Stratification.** Stratified resampling into 10 equal-frequency α buckets ensured balanced coverage of the exponent range.

**Figure 7:**
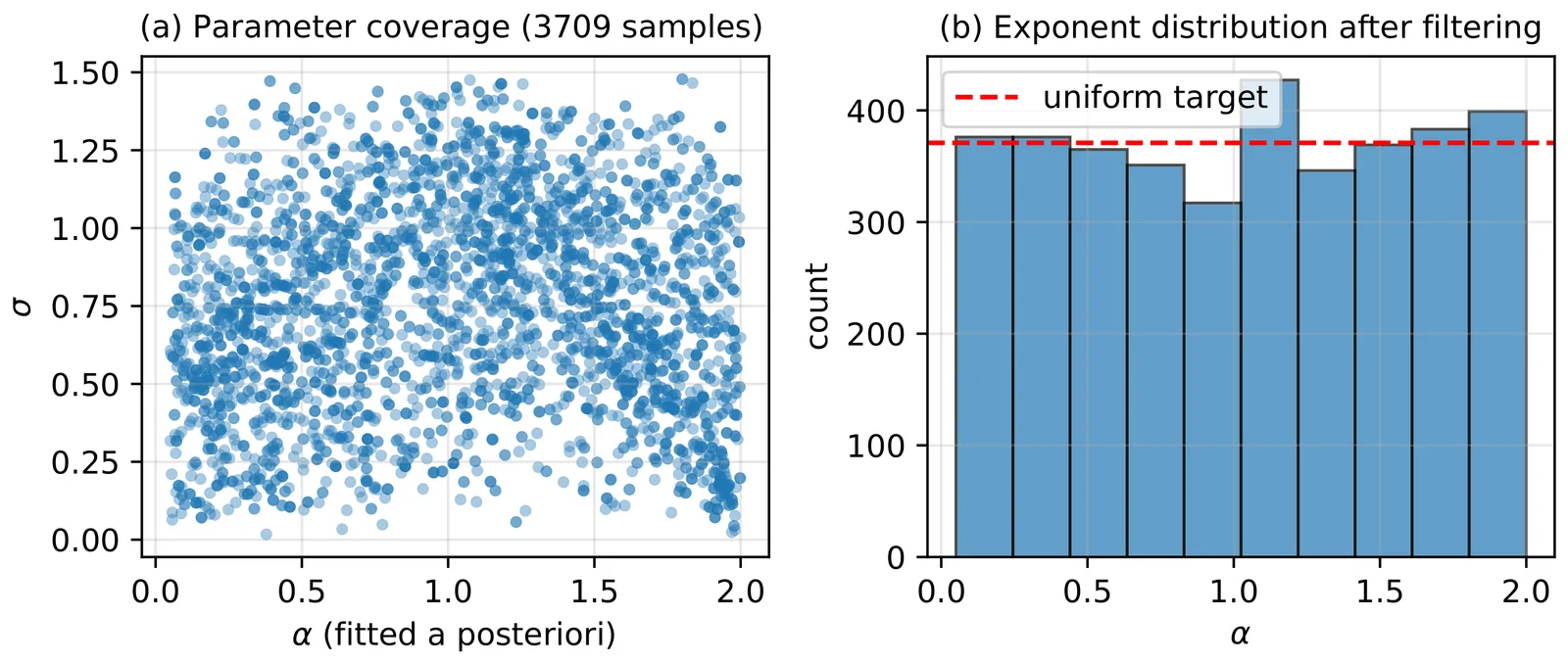
(**a**) Scatter plot of the (α,σ) plane for the 3709 physically admissible samples. Note that α was fitted a posteriori and not sampled. (**b**) Histogram of the α distribution after filtering and stratified resampling into 10 equal-frequency buckets.

The final dataset of 3709 samples was split into training (70%), validation (15%), and test (15%) sets and stratified by the 10 α buckets to preserve distributional balance, yielding 2597 training, 560 validation, and 552 test samples. All statistics were standardized using the training set mean and variance. The parameter coverage and the filtered exponent distribution are visualized in Figure 7.

### 4.2. Implementation Details

The simulator, differentiable PDE solver, and neural network were implemented in PyTorch (version 2.11, CUDA 13) [33]. PyTorch was chosen for three reasons: (1) its automatic differentiation engine allowed the fractional PDE solver to be embedded directly in the training graph, and thus gradients flowed through the L1 scheme to all predicted parameters without manual adjoint derivation; (3) native GPU support accelerated both LSTM training and the batched solver; and (3) its modular ecosystem (optimizers, schedulers, and checkpointing) supported the two-stage training protocol and exact reproducibility through seeded runs. The NumPy/SciPy stack was retained for dataset generation and post hoc analysis. The LSTM encoder had 3 layers with a hidden dimension of 128 and dropout of 0.2. The proxy head had three hidden layers with sizes of 256, 128, and 64, with a dropout of 0.2. Training was performed on the GPU with a batch size of 32. The baseline MLP was a three-layer network with hidden dimensions of [256, 128, 64] that took the flattened statistics as input and directly predicted α via a linear output layer.

All experiments were run on a workstation with an AMD Ryzen 9 9950X 16-core CPU (4.3 GHz), 64 GB of RAM, and an NVIDIA GeForce RTX 5090 D v2 GPU (24 GB) under Windows 11 with Python 3.13, PyTorch 2.11.0+cu130, and NumPy 2.4.6. On this machine, one full 500-step DTQW realization took ≈0.23 s, and training the full FracPINN model (300 epochs, 3709 samples) took ≈10 min on the GPU.

## 5. Results

### 5.1. Main Results

Table 1 summarizes the test set performance of FracPINN and the baseline MLP on the 552-sample test set. FracPINN achieved a mean absolute error (MAE) of 0.214, root mean squared error (RMSE) of 0.318, and coefficient of determination $R^{2}=0.682$. The baseline MLP, trained on the same data without physical constraints, achieved an MAE =0.225, RMSE =0.326, and $R^{2}=0.666$, representing a 5.3% relative improvement of FracPINN in terms of MAE.

**Figure 8:**
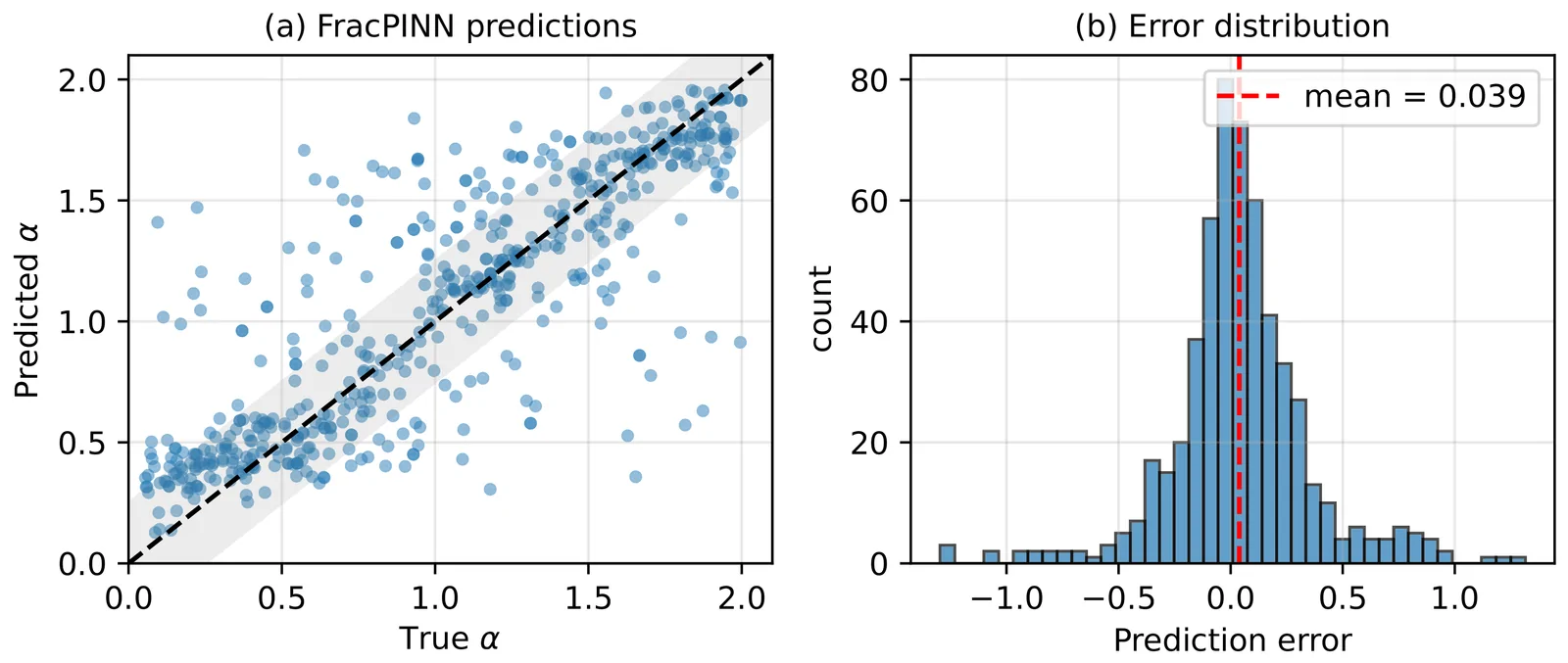
(**a**) Predicted versus true α values on the 552-sample test set. The diagonal dashed line indicates perfect prediction; the shaded region shows ±0.25 error bounds. (**b**) Histogram of prediction errors, with a mean (red vertical line) close to zero, indicating an absence of strong systematic bias.

Figure 8 shows the predicted versus true α values for the 552 test samples. The FracPINN predictions clustered around the diagonal, with a slight bias toward the center of the range due to the physical regularization. The error distribution was approximately Gaussian with a mean of 0.039 and standard deviation of 0.316, confirming the absence of strong systematic bias.

**Figure 9:**
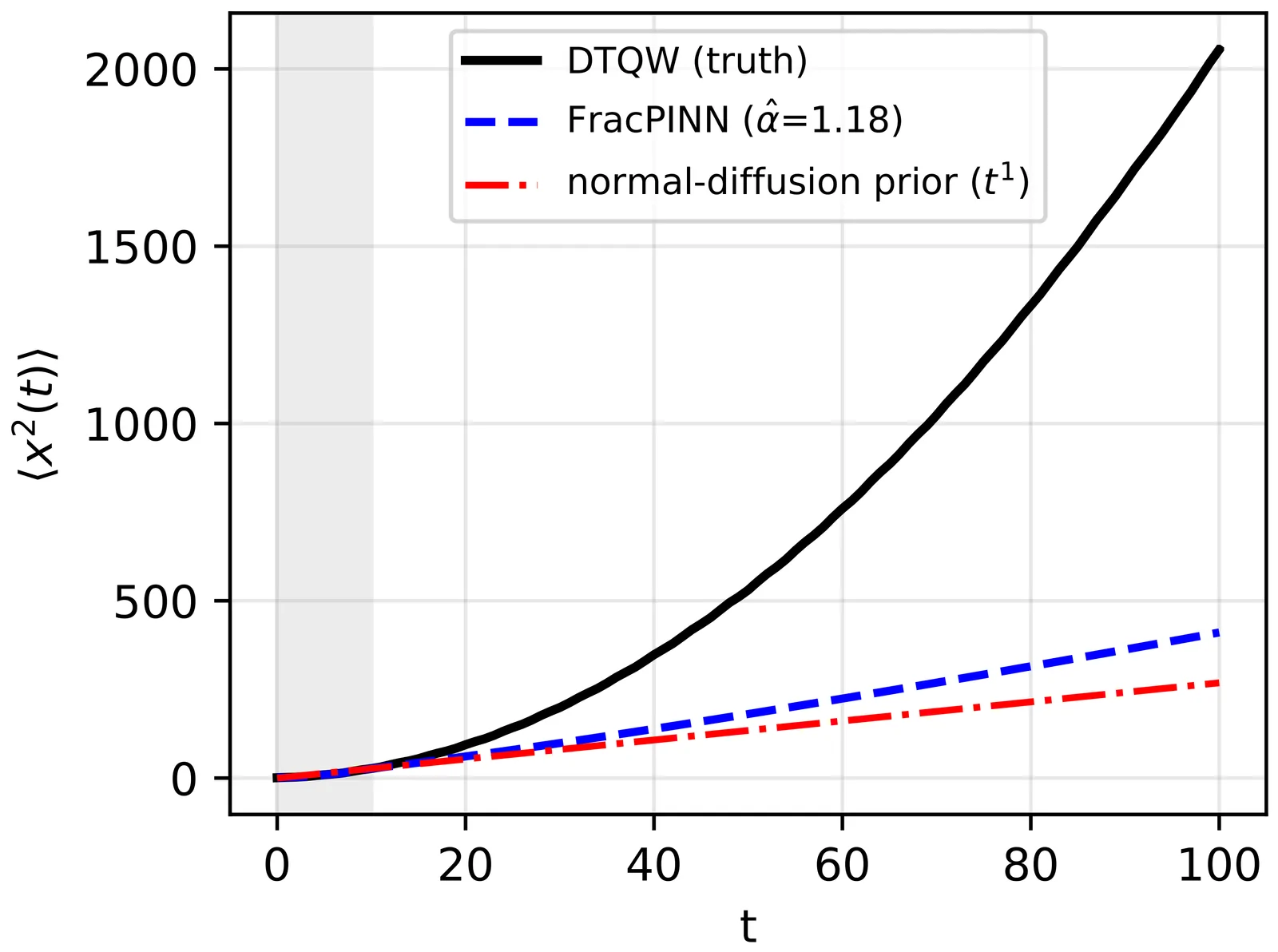
Variance evolution for a representative sample. The FracPINN prediction (dashed blue) closely follows the ground truth (solid black), while the baseline MLP (dash-dotted red) deviates toward normal diffusion scaling ($\langle x^{2}\rangle ∼t$).

The variance evolution predicted by FracPINN closely matched the ground truth for the representative samples, whereas the baseline MLP tended to underestimate the exponent in the super diffusive regime and overestimate it in the sub-diffusive regime (Figure 9). This discrepancy is attributed to the lack of physical constraints in the MLP, which cannot enforce the power-law scaling relationship between variance and time.

### 5.2. Ablation Study

To quantify the contribution of each architectural and loss component, we conducted four ablation experiments: (1) removing the differentiable-solver consistency term and the physical regularization ($\lambda_{\mathrm{PDE}}=0$, $\lambda_{\mathrm{phys}}=0$), (2) removing the data loss ($\lambda_{\mathrm{data}}=0$), (3) reducing the network size to a two-layer LSTM with hidden dimension of 64, and (4) the baseline MLP. Table 2 reports the results.

**Figure 10:**
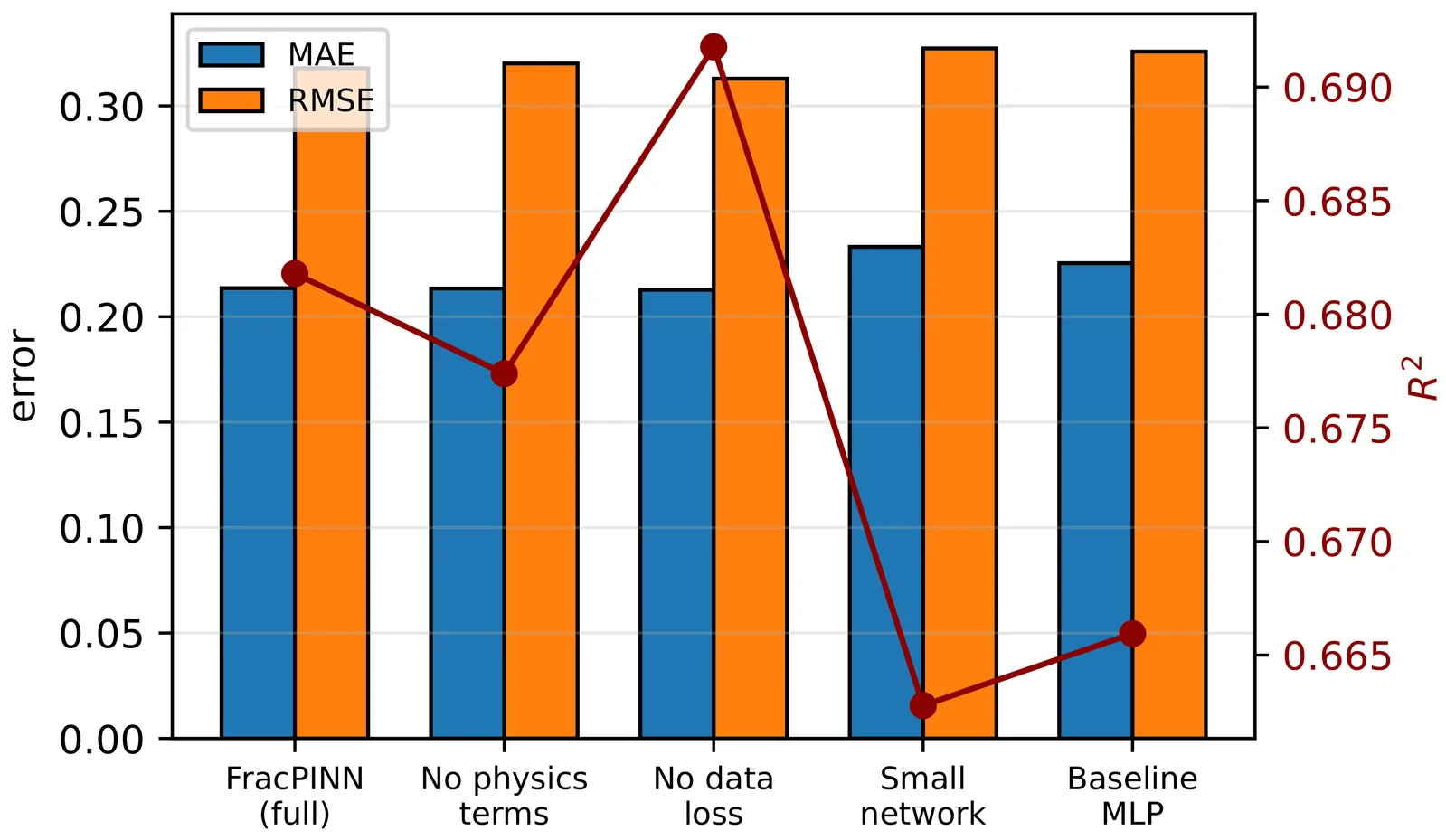
Ablation study comparing FracPINN (full), variants with physics terms removed, data loss removed, reduced network capacity, and the baseline MLP. Left axis: MAE and RMSE; right axis: $R^{2}$.

On the overall test set, the full model, the no-physics ablation, and the no-data ablation performed within about 1% of each other in terms of MAE, while the reduced network and the baseline MLP were clearly worse (9% and 5% degradation, respectively). This shows that the physics-based parameterization—predicting $\left(\beta ,\gamma ,\tau_{m},D_{0}\right)$ through the constrained head with the power-law proxy—already provided the main in-distribution benefit and that the solver-level physics terms did not degrade performance. Crucially, the solver-level physics terms yielded their largest gain where it was physically expected; in the sub-diffusive regime, the full model improved the MAE by 4.1% over the no-physics ablation and by 13.5% over the baseline MLP (Table 3), because the memory–kernel dynamics discriminated between trapped and merely slow-spreading. We therefore revised our earlier, stronger claim that the physics terms dominate the overall accuracy: their contribution is regime-specific, which we now state explicitly. The ablation comparison is summarized visually in Figure 10.

### 5.3. Regime Analysis

**Figure 11:**
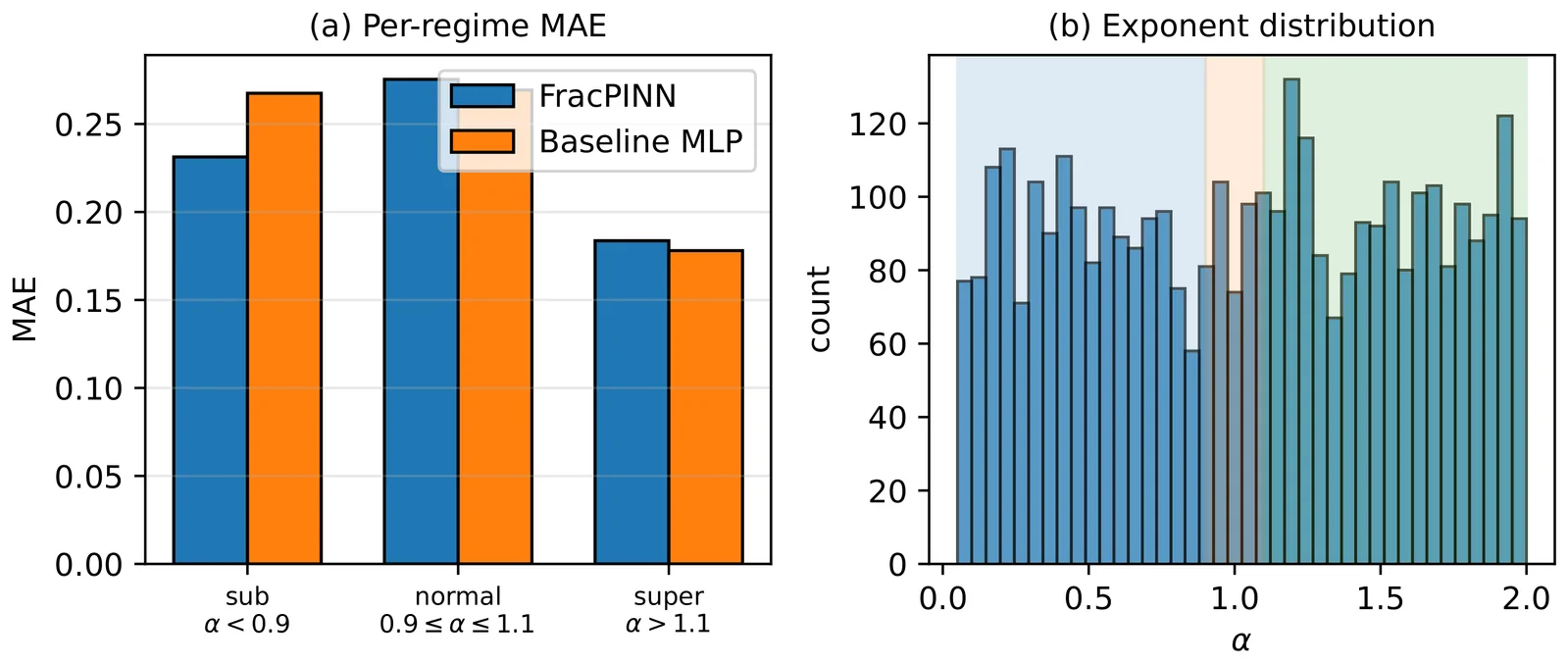
(**a**) Performance metrics (MAE and RMSE) by diffusion regime: sub-diffusion (α<0.9), normal (0.9≤α≤1.1), and super diffusion (α>1.1). (**b**) Histogram of the α distribution across the dataset, colored by regime.

We partitioned the test set into three diffusion regimes based on the true α: sub-diffusion (α<0.9), normal diffusion (0.9≤α≤1.1), and super diffusion (α>1.1). Table 3 reports the per-regime metrics. FracPINN achieved its clearest advantage in the sub-diffusive regime (MAE =0.231 vs. 0.267 for the baseline MLP, a 13.5% improvement, and 0.241 for the no-physics ablation), where the non-Markovian memory was strongest and the physics-informed terms provided genuine discrimination. In the super-diffusive regime, the model performed comparably to the baseline (MAE =0.184 vs. 0.178), and in the narrow normal regime (α∈[0.9,1.1], only 60 test samples) both models showed their largest relative errors, as small absolute deviations were significant against the compressed label range. The within-regime $R^{2}$ values were not informative here, because the label variance inside each narrow regime slice was small; we therefore report the MAE and RMSE only. The per-regime comparison is visualized in Figure 11.

The baseline MLP exhibited a larger degradation in the sub-diffusive regime (MAE =0.267 versus 0.231 for FracPINN), consistent with the hypothesis that physics-informed regularization is most beneficial when the dynamics deviate strongly from the classical limit.

### 5.4. Long-Term Extrapolation

**Figure 12:**
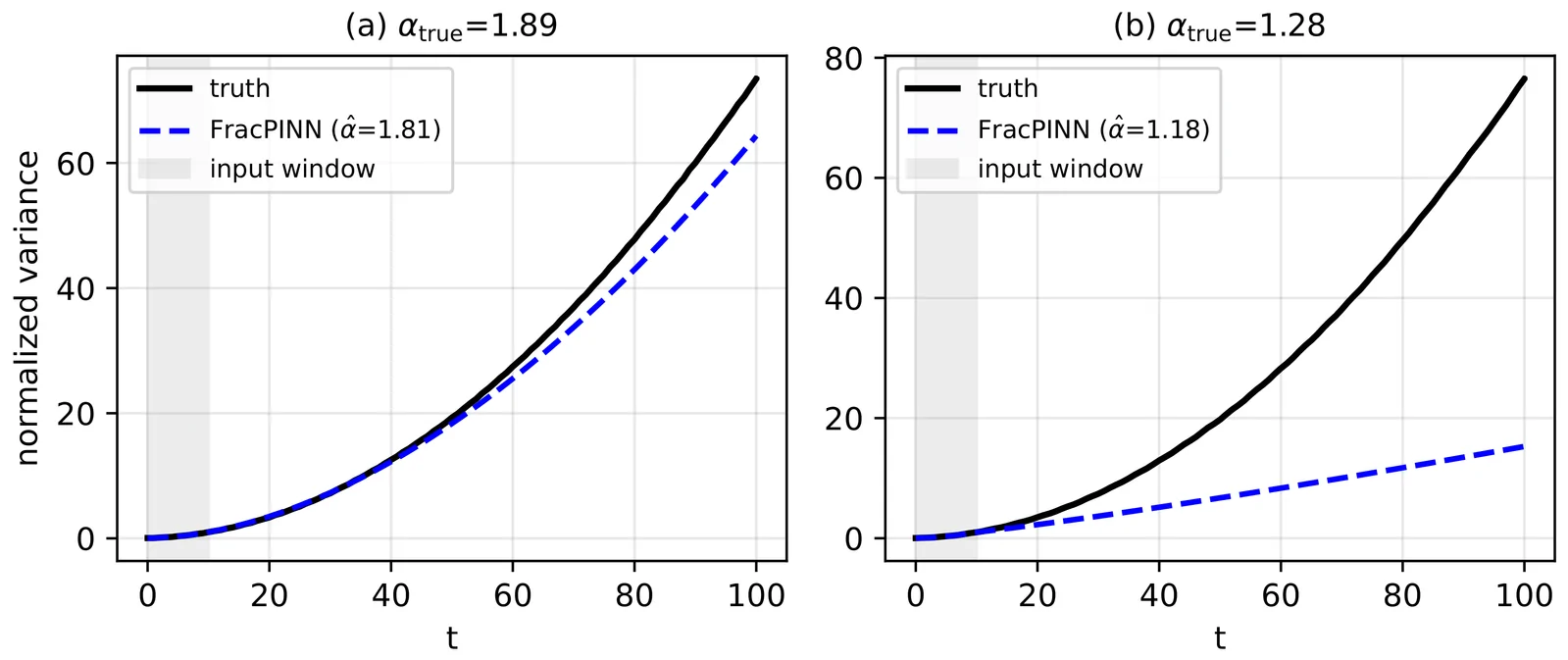
Long-term extrapolation test on two held-out simulations. Training on early-time statistics (t≤10, shaded region), FracPINN extrapolated the variance curve to t=100 (dashed) against the ground truth (solid). (left) A super-diffusive in-distribution case ($\alpha_{\mathrm{true}}=1.89$, $\hat{\alpha }=1.81$). (right) A case closer to the edge of the training range, where the error was larger.

A frequently advertised benefit of physics-informed models is extrapolation beyond the training window. We assessed this honestly with six held-out DTQW simulations (500 steps each) that were not part of the dataset. For each simulation, the model received only the early statistics (t≤10) and predicted the exponent $\hat{\alpha }=2\hat{\beta }$. The extrapolated variance curve $v\left(t\right)=v\left(10\right)\;{\left(t/10\right)}^{\hat{\alpha }}$ was then compared with the true simulated variance up to t=100 (10 times the input window). For the simulations lying inside the filtered training range, the predicted exponents were accurate to $|\hat{\alpha }-\alpha_{\mathrm{true}}|\;\approx 0.03$–0.12 (e.g., $\alpha_{\mathrm{true}}=1.89$ versus $\hat{\alpha }=1.81$), and the extrapolated variance tracked the true curve with a relative error of ≈12% at t=100 (Figure 12). For strongly trapped or transient realizations outside the training range, however, power-law amplification of the exponent error over a decade in time produced substantially larger curve deviations. We therefore state the extrapolation capability with care: the surrogate yields reliable exponent estimates and useful medium-horizon predictions within the training distribution, but it is not a substitute for simulation far outside it.

### 5.5. Computational Efficiency

Once trained, FracPINN evaluated a single sample in ≈0.33 ms on the CPU (AMD Ryzen 9 9950X), whereas one full 500-step DTQW realization required ≈0.23 s on the same machine, a speedup of ≈$7\times 10^{2}$ per realization. For ensemble studies that averaged over M=50 realizations per parameter set, the simulation cost rose to ≈11 s, corresponding to a speedup of ≈$3\times 10^{4}$; batched GPU inference widened the gap further. Training the full model took ≈10 min on the RTX 5090 GPU for 3709 samples and 300 epochs. The inference speedup makes the surrogate suitable for interactive parameter estimation and Bayesian calibration workflows.

## 6. Discussion

Our results demonstrate that fractional physics-informed neural networks can effectively serve as surrogate models for quantum random walk parameter estimation, bridging the gap between microscopic quantum simulation and mesoscopic fractional transport theory. The key insight is that coarse-grained statistical observables, variance, entropy, kurtosis, and spatial correlations, contain sufficient information to infer the effective anomalous diffusion exponent, provided the inference is constrained by the appropriate fractional PDE.

The ablation study revealed a nuanced trade-off between data-driven and physics-driven learning. In the normal diffusion regime, the data loss alone was nearly sufficient, reflecting the fact that the variance scaling was approximately linear and easily captured by regression. In the sub-diffusive regime, however, the physics terms became important; without them, the network tended to predict exponents near the population mean, failing to capture the slow, memory-trapped spreading characteristic of strong non-Markovianity. This is consistent with the observation in [23] that anomalous diffusion classification benefits from physical priors when the signal is weak relative to noise.

**Figure 13:**
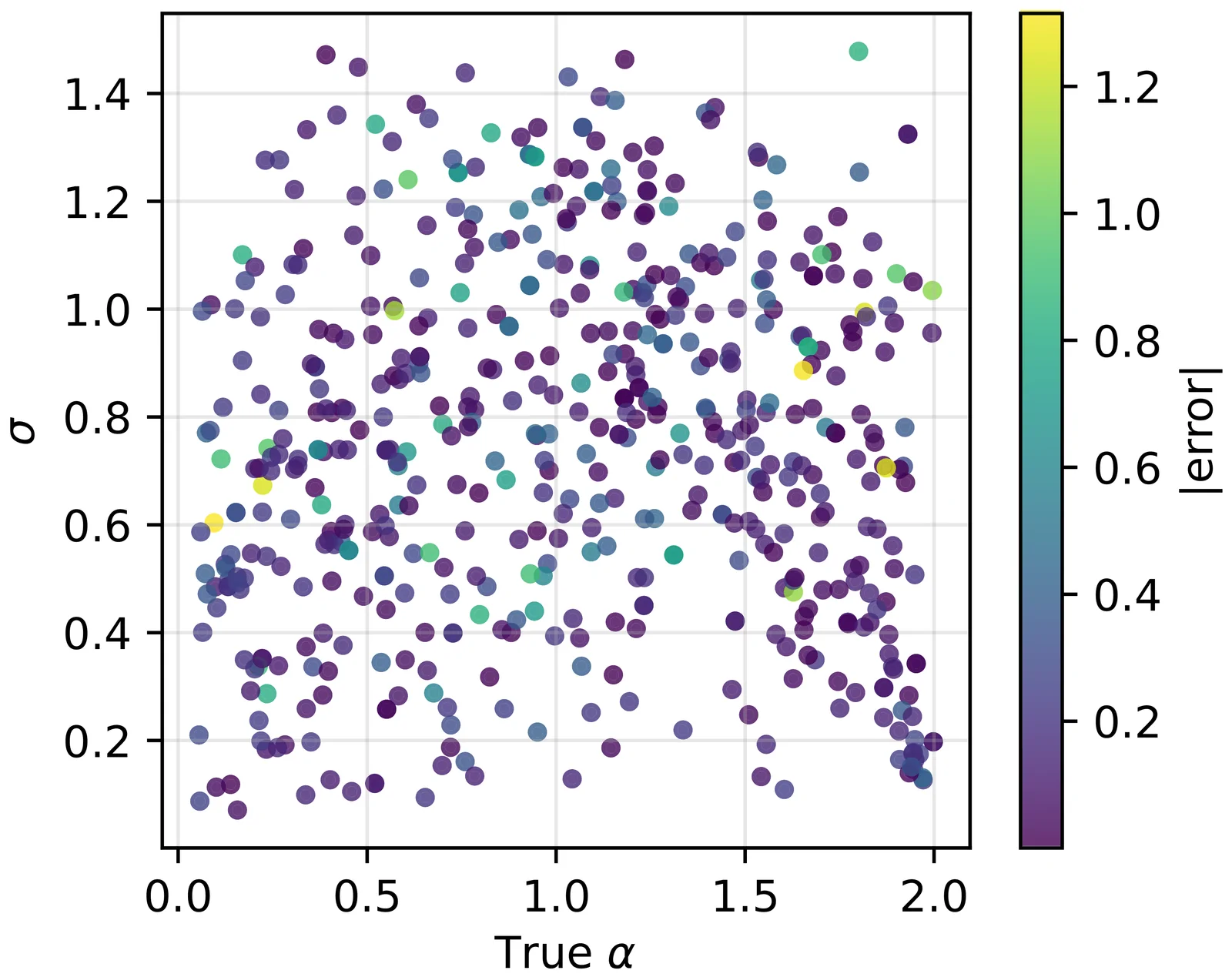
Prediction error (MAE) landscape as a function of the true parameters (α,σ). Errors are smallest near the center of the parameter space where the training data are most concentrated.

Several limitations of the current framework warrant discussion. First, the fractional PDE in Equation (5) is an effective model; it does not capture microscopic quantum coherence effects such as revival and partial localization that may occur at specific parameter values. Future work could incorporate a Schrödinger-like term or a nonlocal potential to account for these effects. Second, the PDE solver is currently one-dimensional; extending it to two or three spatial dimensions would enable the study of more complex lattice geometries and graph structures [34]. Third, the memory kernel is parameterized as a simple exponential; a more flexible non-parametric kernel could be learned jointly with the neural network, as suggested by recent work on kernel discovery with neural operators [14]. Fourth, in the pure Caputo limit, the fractional diffusion equation sustains at most linear MSD growth, and thus the strongly super-diffusive and ballistic regimes are represented through the Hurst-like power-law proxy rather than through the PDE solution itself; a fractional diffusion-wave formulation (derivative order in (1,2]) would cover these regimes directly and is a natural extension. Fifth, the explicit L1 solver on the unit grid loses accuracy for deep sub-diffusion (β≲0.5; see Section 3); implicit schemes or adaptive refinement would remove this restriction. Sixth, each dataset sample is a single noise realization, and thus the per-sample statistics carry realization noise that an ensemble average would reduce at additional computational cost. Figure 13 shows the prediction error landscape across the (α,σ) parameter space, highlighting the region of highest accuracy near the center of the training distribution.

From an experimental perspective, the framework is directly applicable to trapped ion quantum walk platforms, where the coin noise can be engineered by coupling the ion to an engineered bath with a tunable correlation time [5]. In such settings, the coarse-grained statistics could be extracted from fluorescence images, and the FracPINN could provide real-time feedback for bath parameter calibration. Photonic implementations [13] are another promising venue, as the walker’s spatial distribution is naturally accessible via array detectors.

Finally, we note that the surrogate modeling approach developed here is not limited to quantum walks. The same framework could be applied to classical anomalous diffusion in complex media [25], fractional viscoelasticity [20], and transport in porous materials [35], or wherever the governing dynamics are characterized by memory and fractional derivatives.

## 7. Conclusions

We introduced FracPINN, a fractional physics-informed neural network that learns to infer the effective anomalous diffusion exponent and fractional transport parameters from coarse-grained statistical observables of non-Markovian discrete-time quantum walks. By embedding a differentiable Caputo derivative PDE solver into the neural network and training with a multi-component loss function whose physics-informed terms are strictly label-free, the model achieved accurate predictions across sub-diffusive, normal, and super-diffusive regimes. On a dataset of 3709 physically admissible DTQW samples, FracPINN attained an MAE of 0.214 and $R^{2}=0.682$, outperforming a purely data-driven baseline by 5.3% in terms of MAE and by 13.5% in the memory-dominated sub-diffusive regime. Once trained, the model provided inference in fractions of a millisecond, enabling real-time parameter estimation for quantum simulation and transport characterization. We plan to extend the framework to higher dimensional lattices, nonparametric memory kernels, fractional diffusion-wave dynamics for the ballistic regime, and experimental data from trapped ion and photonic platforms.

## Figures and Tables

**Table 1:** Testset performance comparison.

Model	MAE	RMSE	$R^{2}$
FracPINN (full)	0.214	0.318	0.682
Baseline MLP	0.225	0.326	0.666

**Table 2:** Ablation study results.

Experiment	MAE	RMSE	$R^{2}$
FracPINN (full)	0.214	0.318	0.682
No physics terms	0.213	0.320	0.677
No data loss	0.213	0.313	0.692
Small network	0.233	0.327	0.663
Baseline MLP	0.225	0.326	0.666

**Table 3:** Performance by diffusion regime.

Regime	FracPINN MAE (RMSE)	Baseline MAE (RMSE)	*n*
Sub-diffusion (α<0.9)	0.231 (0.334)	0.267 (0.365)	231
Normal (0.9≤α≤1.1)	0.275 (0.357)	0.269 (0.360)	60
Super diffusion (α>1.1)	0.184 (0.293)	0.178 (0.276)	261

## Data Availability

The original contributions presented in this study are included in the Supplementary Materials archive. Further inquiries can be directed to the corresponding authors.

## Supplementary Materials

The following supporting information can be downloaded at https://www.mdpi.com/article/10.3390/e28080844/s1. Data S1: qw_dataset_physical.
